# Gross on the Urinary Organs

**Published:** 1851-08

**Authors:** 


### GROSS ON THE URINARY ORGANS.

The work promised sometime since by Professor Gross, on the "Diseases and Injuries of the Urinary Bladder, the Prostate Gland, and the Urethra," has just been issued in Lea & Blanchard's best style, and through the politeness of the author we have had an opportunity of looking through its pages. In advance of the full notice which we propose to give of its contents in our next number, we take pleasure in expressing the opinion, that no more valuable work on surgery has appeared in our country. It is a work which was much needed, and has been prepared with a care and a judgment which will enhance the reputation of the distinguished author. There is not in the language a monograph so full, complete, and satisfactory on the interesting class of affections of which it treats. D. W. Y.

